# Concentration, motivation, activity, and subjective fatigue in patients with single-sided deafness

**DOI:** 10.1017/S0022215124001002

**Published:** 2024-11

**Authors:** Emre Gürses, Samet Kılıç, Bünyamin Çıldır

**Affiliations:** 1 Department of Audiology, Faculty of Health Science, Hacettepe University, 06100 Ankara, Turkey; 2 Department of Audiology, Faculty of Health Science, Trakya University, Edirne, Turkey; 3 Language and Speech Therapy Department Health Sciences Faculty, Ankara Yildirim Beyazit Üniversity, Ankara, Turkey

**Keywords:** unilateral deafness, chronic fatigue syndromes, attention, motivation, physical activity

## Abstract

**Objective:**

To evaluate four dimensions of fatigue, including subjective fatigue severity, concentration problems, reduced motivation, and activity in patients with single-sided deafness.

**Methods:**

Following audiological assessment, the Checklist Individual Strength scale and Montreal Cognitive Assessment were performed on 41 adults with single-sided deafness and 41 sex-matched adults with normal bilateral hearing in the study group and control group, respectively. Subjective fatigue severity, concentration, motivation, activity level and cognitive performance were analysed between and within groups.

**Results:**

Individuals with single-sided deafness exhibited reduced concentration and motivation; however, their activity level was average. Subjective fatigue symptoms were more prevalent in individuals with single-sided deafness than in control participants. The concentration problem was related to decreased cognitive performance.

**Conclusion:**

This study revealed negative somatic consequences of single-sided deafness. Self-perceived fatigue is likely underestimated in this population due to the limited studies reported in the literature. Further studies should focus on counselling, follow up and hearing rehabilitation concerning ameliorating fatigue.

## Introduction

Single-sided deafness (SSD) is determined by normal hearing thresholds in one ear and severe hearing loss in the other. Previous studies have shown that individuals with single-sided deafness lack many physiological processes. The most significant difficulty for individuals with single-sided deafness is decreased speech intelligibility in noisy environments.^1^ Compared to normal hearing, in the presence of single-sided deafness, individuals with SSD should have a signal-to-noise ratio of at least 2–9 dB more in order to distinguish speech from noise.^2^ Moreover, the localisation ability of the sound source in the horizontal plane has deteriorated. While the ability to localise the sound source in children with normal hearing shows a margin of error between 4° and 6°,^3^ the average error margin for localisation increases to 28° in individuals with single-sided deafness.^4^ Although localisation ability is not impaired in the vertical plane, it has been reported that there is a decrease in acuity compared to individuals with normal bilateral hearing.^5^ In addition, individuals with single-sided deafness also lack the skills of lateralisation, precedence effect, virtual auditory space localisation, diffraction effect of the head, binaural beats, release from masking, unmasking, auditory stream segregation and summation.^6^ Decreases in auditory distance perception skills^7^ and dereverberation have also been reported.^8^ Poor auditory processing abilities could cause daily life quality loss and academic challenges related to more deficient motivation, concentration, and activity, leading to increased subjective fatigue.

In a study conducted with school-age children with single-sided deafness, individuals with single-sided deafness were described as aggressive and distracted.^9^ In the same survey, it was stated that although most of the children were in their first years of school, 35 per cent of them repeated the grade. This rate is ten times higher than their peers with normal hearing.

Previous studies showed that individuals with single-sided deafness have stress and increased fatigue due to decreased cognitive skills in hearing effort.^10,11^ A functional magnetic resonance imaging study about auditory tasks stimulated by narrow-band noise showed alterations of activation in hearing-related areas and attention networks in single-sided deafness.^12^ Moreover, alterations in attention and associated visual areas were found in a similar methodology but with different stimulation (speech-in-noise).^12^

Considering anatomical, cognitive, segmental, and supra-segmental changes in single-sided deafness, we proposed that patients with single-sided deafness might have somatic complaints such as poor concentration, motivation, activity, and subjective fatigue that affect daily living activities and result in chronic fatigue syndrome. Chronic fatigue is a difficult to define, complicated subjective sensation that lasts at least six months and is characterised by unmanageable tiredness, diminished energy and a sense of burnout.^13^ Chronic fatigue differs from the acute short-term fatigue, muscle fatigue and sleepiness we experience daily. Furthermore, fatigue is subjective and has no clear objective markers.^14^ Thus, subjective scales are preferred in evaluation.

Checklist Individual Strength is one of the most frequently used, valid, and reliable tools to evaluate fatigue.^14^ This scale evaluates fatigue from four aspects: subjective experience, decrease in motivation, reduction in activity, and decrease in concentration.^14^ Studies evaluating hearing loss and fatigue are few in the literature. A study of 149 adults with sensorineural, mixed and conductive hearing loss using the multidimensional fatigue symptom inventory short form showed increased subjective fatigue perception.^15^ However, no statistically significant regression was obtained between the same study's hearing-loss level and fatigue severity. It has been determined that the studies evaluating subjective fatigue in individuals with unilateral total hearing loss could be more extensive in the literature. Therefore, our study aimed to assess chronic fatigue in individuals with single-sided deafness. The study's second aim was to evaluate whether there is a relationship between the duration of deafness and Checklist Individual Strength. The study's third aim was to assess the correlation between the cognitive scores of individuals with single-sided deafness and chronic fatigue.

## Materials and Method

### Study design

A cross-sectional design was used for the current study. Ankara Yıldırım Beyazıt University Non-Interventional Clinical Research Ethics Board gave its approval for this study (2021–90). Written informed consent was obtained from all individual participants included in the study.

### Participants

We included 41 participants with single-sided deafness and 41 gender-matched participants with normal hearing. Participants were recruited from the audiological clinic and social environment. Individuals with single-sided deafness were the study group, and individuals with normal hearing were the control group.

Inclusion criteria in the study group were (1) ages between 18 and 55 years, (2) unilateral total hearing loss lasting at least one year, (3) hearing thresholds ≤ 20 dB in the contralateral ear (125–8000 Hz), (4) having a speech identification score of 92 per cent or more in the contralateral ear, and (5) having a score of 21 or higher on the Montreal Cognitive Assessment test. Inclusion criteria in the control group were (1) ages between 18 and 55 years, (2) bilateral hearing thresholds ≤ 20 dB, (3) having a speech identification score of 92 per cent or more in both ears, (4) no tinnitus, (5) no neurological problems, and (6) a score of 21 or higher on the Montreal Cognitive Assessment test.

Exclusion criteria were (1) conductive and mixed hearing loss; (2) having bilateral hearing loss; (3) unilateral mild, mild–moderate, or severe hearing loss; (4) having physical and emotional disorders that may affect test results; (5) having single-sided deafness using a cochlear implant in an ear with hearing loss; and (6) having a history of neurological problems. Moreover, any diagnoses that could cause chronic fatigue (diabetes, musculoskeletal system, psychiatric and neurological disorders, alcohol or substance abuse, anorexia, obesity) except for acoustic neuroma, which is known to be related to single-sided deafness, were also excluded. The demographic information of the participants is given in Table 1.

**Table 1.** Demographic information of the participants with single-sided deafness (SSD); *N* = number; NA = not applicable

SSD	Onset of Hearing Loss (N)					Mean Hearing Loss Duration (years)	
	Congenital (24%)		Acquired (73%)		NA (3%)		
	Female	Male	Female	Male		Female	Male
Right Ear	6	3	9	7	1	19.71	13.71
Left Ear	0	1	7	6	1	20.00	27.88
Subtotal	6	4	16	13	2	19.90	21.27
Total	41					20.45	

### Audiological Assessment

Hearing thresholds of all participants were performed using ER.3A insert headphones (Etymotic Research, Elk Grove Village, Illinois) at all octave frequencies from 125 Hz to 8000 Hz in a double-walled sound-attenuating booth (IAC Acoustics, Naperville, Illinois) according to a modified Hughson–Westlake procedure. In addition, we performed a bone-conduction threshold test at 500–4000 Hz using a RadioEar B-71 bone vibrator (RadioEar, Middelfart, Denmark). A word recognition test was assessed in quiet conditions using 25 phonetically balanced monosyllabic words with the same audiometry equipment. Pure tone means (0.5–4 kHz), ≥ 85 dB hearing level, and ≤ 20 dB hearing level at all octave frequencies in the contralateral ear included in the study group, and when ≤ 20 dB hearing level at all octave frequencies in both ears in the control group. Word discrimination scores of 92 per cent and above in the clinically normal hearing ear for the control group and both ears for the study group were included.

### Fatigue assessment

Fatigue assessment of the participants was made using the Checklist Individual Strength questionnaire.^16^ The questionnaire includes 20 items to measure four aspects of fatigue. Items of the questionnaire are distributed as follows: fatigue severity (8 items), concentration (5 items), motivation (4 items), and physical activity (3 items). A seven-point Likert scale was used for each item. Increased scores show elevated levels of fatigue, concentration problems, low motivation, and weak levels of physical activity. According to evidence-based results, a score of 35 or higher on the fatigue-severity subscale indicates severe experiences of fatigue^14^ In addition, a score between 27 (healthy adults’ mean score plus one standard deviation) and 35 shows a validated heightened experience of fatigue^17^

### Montreal Cognitive Assessment

Montreal Cognitive Assessment is used to screen for cognitive impairment and assess different cognitive functions.^18^ These cognitive functions are “visuospatial/executive,” “naming,” “memory,” “attention,” “language,” “abstraction” and “delayed recall and orientation (to time and place).” The highest score that can be obtained from the test is 30. A Montreal Cognitive Assessment score of 21 and above was considered normal and included in the study. One point was added to the scores of participants with less than 15 years of education.

### Statistical analyses

All data were analysed using the SPSS 24 (IBM Corporation, Armonk, New York) program. The assumption of normal distribution was analysed via visual (histograms, probability plots) and analytical (Kolmogorov–Smirnov test) techniques. Means and standard deviations were used to present the results of descriptive analyses. Differences between single-sided deafness and normal hearing groups were compared using an independent samples *t*-test. The statistical significance level was set at *p* < 0.01 because of multiple pairwise comparisons. We used Cohen's *d* for size-effect measurement. Calculations were done using (M_2_−M_1_)/√((SD_1_^2^ + SD_2_^2^)/2) formula. Correlations between the duration of deafness and chronic fatigue levels were assessed using the Pearson correlation coefficient.

## Results

### Participants

Forty-one participants with single-sided deafness with a mean age of 37.09 ± 11.27 years and 41 gender-matched participants with normal bilateral hearing with a mean age of 34.75 ± 6.79 years were included in the study (see Table 1). The aetiologies of the study group were eight participants with a congenital malformation, seven participants with sudden sensorineural hearing loss, four participants with mumps, four participants with schwannoma, and two participants with Ménière's disease, followed by one participant with head trauma, labyrinthitis, cholesteatoma, iatrogenic, large vestibular aqueduct syndrome, ototoxicity during pregnancy, premature birth, three participant who remains unspecified but has a history of familial hearing loss, and six participants deemed unclear.

We found no differences between participants with single-sided deafness and normal bilateral hearing regarding age [*t* (80) = 1.138, *p* = 0.25]. However, although all participants had more than the stipulated cut-off points (> 21), the normal bilateral hearing group had statistically better results in the Montreal Cognitive Assessment scores [*t* (75) = −3.509, *p* = 0.001], Cohen's *d* = 0.80.

### Fatigue severity in patients with single-sided deafness

Subjective fatigue subscale results of the single-sided deafness group were assessed based on severe experiences of fatigue (cut-off score > 35) and heightened experiences of fatigue (cut-off score = 27–35). We found that 13 (31.7 per cent) participants had severe subjective fatigue, whereas 17 (41.4 per cent) had a heightened experience of fatigue. No gender differences were found in the total score of Checklist Individual Strength [*t* (38.9) = 0.859, *p* = 0.396], Cohen's *d* = 0.26. The correlation between duration of deafness and Checklist Individual Strength score showed that chronic fatigue was not related to the duration of deafness (*p* = 0.68, *R* = −0.085). There was a statistically significant, moderate, negative association between Montreal Cognitive Assessment and concentration scores of the participants with single-sided deafness (*p* = 0.001, *R* = −0.528).

### Chronic fatigue comparison

**Figure 1.**
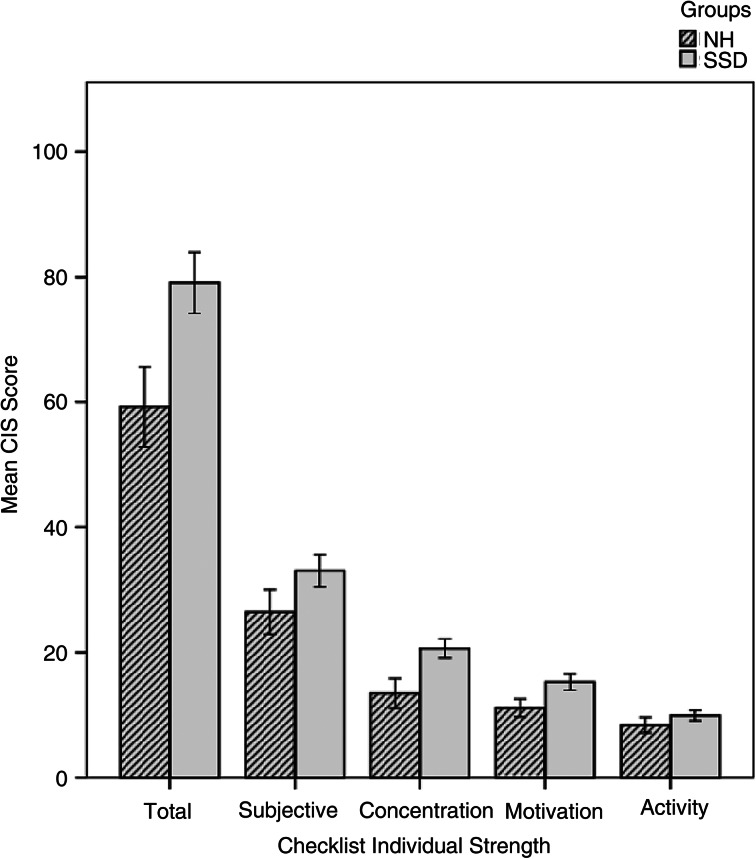
Chronic fatigue ratings between participants with single-sided deafness (SSD) and normal bilateral hearing (NH). Error bars represent ± 2 standard errors. CIS = Checklist Individual Strength.

The total score and four subscales, including subjective fatigue, concentration, motivation, and activity, were compared between the two groups (Table 2). Participants with single-sided deafness had greater subjective fatigue, less concentration and less motivation. However, activity scores revealed that there was no statistical significance between the two groups (Figure 1).

**Table 2.** Comparison of Checklist Individual Strength (CIS) score between single-sided deafness (SSD) and normal bilateral hearing (NH) groups; SD = standard deviation; *df* = degrees of freedom

CIS	SSD		NH		*t*	*df*	*p*	Cohen's *d*
	Mean (SD)	Min–Max	Mean (SD)	Min–Max				
Total	79.0 (15.4)	44–115	59.5 (20.7)	20–98	4.8	80	< 0.001	1.0
Subjective Fatigue	33.0 (8.0)	18–56	26.4 (11.1)	8–55	3.0	80	0.003	0.6
Concentration	20.6 (4.9)	9–33	13.44 (7.4)	5–32	5.1	69	0.008	1.1
Motivation	15.2 (4.1)	7–22	11.1 (4.5)	4–21	4.3	80	< 0.001	0.9
Activity	9.9 (2.5)	3–15	8.3 (3.9)	3–15	2.1	68	0.03	0.4

## Discussion

There is ongoing debate regarding the pathogenesis of chronic fatigue syndrome. However, an increasing number of studies have indicated that many patients have aberrant biological processes. In this study, we aimed to assess chronic fatigue in patients with monoaural hearing and reveal the associations between hearing-related difficulties and subjective feelings of fatigue, concentration problems, reduced motivation and reduction of activities.

We found that patients with single-sided deafness had more fatigue than patients in the normal hearing group. However, fatigue results were not correlated with duration of hearing loss. Instead, the aetiology of the participants led to fatigue levels. Acoustic schwannomas and iatrogenic conditions were associated with our patient population's most severe fatigue level. Chronic cancer-related fatigue is well known in previous studies.^19^ Although our results comply with earlier findings in patients with cancer, 31.7 percent of participants with single-sided deafness had severe chronic fatigue levels in our patients. Since we had only two patients with schwannomas, aetiologies could not solely explain our results. Listening difficulties could be one of the main contributors.

The cognitive resources necessary to accomplish a listening task are called listening effort. Individuals with hearing loss need more listening effort, even when utilising hearing aids or cochlear implants.^20^ People who put forth a lot of “ineffective” listening efforts are more likely to report unfavourable effects on their social and emotional lives.^21^ Even hearing-related difficulties without hearing loss, such as tinnitus, were found to require an extra effort to listen.^22,23^ Therefore, hearing loss and hearing-related difficulties could be related to listening fatigue due to the listening effort. We found that the total fatigue level in the single-sided deafness population is higher than in the normal bilateral hearing population.

Although individuals with monoaural hearing discern well in speech intelligibility scores in quiet settings, individuals with single-sided deafness have difficulties in environments with background noise.^24^ Moreover, poorer suprasegmental auditory processing ability could force individuals to exert more effort because of auditory deprivation.^25^ Total fatigue results could be explained by exerting more cognitive effort to discern speech in noisy settings by individuals with single-sided deafness.

According to McShefferty *et al*.,^26^ listening fatigue is characterised as “extreme tiredness caused by [unrewarding] effortful listening.” Therefore, the Montreal Cognitive Assessment was performed to understand the contributor factor of higher fatigue in the single-sided deafness group. We found poor performance in patients with single-sided deafness compared to patients with normal bilateral hearing. Furthermore, we found that poor cognitive performance caused debilitating chronic fatigue in individuals with single-sided deafness.

Our study supports previous findings that cognitive performance is poorer in individuals with single-sided deafness than in individuals with normal bilateral hearing. We performed a correlation analysis between the Montreal Cognitive Assessment and Checklist Individual Strength scores to understand the relationship between cognitive capacity and fatigue. We previously excluded individuals with less than 21 Montreal Cognitive Assessment points, which indicates mild cognitive impairments for both groups. Although Montreal Cognitive Assessment is a screening assessment, it was found that the concentration subscale and Montreal Cognitive Assessment results were statistically significant. Lower concentration could be a result of straining to hear, especially in noisy environments for extended periods of time. On the other hand, poor concentration might be a behavioural consequence of electrophysiological activity alteration, especially in attention networks.^12^ Our population had no experience with hearing amplification. Further studies should focus on hearing amplification benefits for individuals with concentration difficulties and single-sided deafness.

Social and psychological wellbeing was found to be affected according to three group interviews using the critical incident technique in a study with eight adults with single-sided deafness.^10^ Limitations on activities and engagement, including withdrawal from and within contexts, were social repercussions of single-sided deafness. The psychological effects mentioned by participants included embarrassment related to the social stigma associated with hearing loss and diminished confidence and belief in their ability to participate.

Our findings support and amplify the negative consequences of single-sided deafness. Poorer concentration and motivation findings in our study may be the underlying factors for these results. According to Hetu *et al*.,^27^ tiredness from more intense listening efforts may induce a hearing-impaired individual to “give up” on interpreting speech, which could lead to communication withdrawal. Therefore, lower motivation results may be due to tiredness and negatively contribute to communicative disengagement in individuals with single-sided deafness.

- People experiencing hearing loss on one side tend to report higher subjective fatigue symptoms than those with normal hearing
- People with single-sided hearing loss often exhibit decreased concentration and motivation despite maintaining an average activity level
- A noticeable association exists between reduced concentration and a decline in cognitive performance in individuals with single-sided hearing loss
- Because hearing, listening and attention are distinct but interrelated processes, without formal cognitive and objective fatigue assessment, definitive conclusions may not be yielded

Aging affects listening effort,^28^ temporal processing,^29^ and motor and cognitive performance,^30^ so age differences between groups could likely affect fatigue results. Therefore, we compared age differences in the studied groups. Since we didn't find any discrepancies, we concluded that statistically significant differences are not related to age differences but to hearing loss. However, it should be borne in mind that fatigue is a multi-modal assessment, and there are many contributors to fatigue, such as daily routine, work, and social representatives. Although we found poorer motivation, concentration, and fatigue, we found no statistical differences in activity levels between the two groups, which supports the idea about multimodal inputs of fatigue level.

### Study limitations and future directions

Although the poor cognitive functions we revealed are compatible with previous studies, the results must be considered within the limitations found. To show cognitive performance differences, we screened participants using the Montreal Cognitive Assessment test. However, we could have attained more accurate conclusions with detailed cognitive assessments (such as working capacity). It is important to emphasise that hearing and listening, as well as attention, are distinct yet closely interconnected processes. Differentiating between these processes is often challenging in children and even more so in adults, where cognitive or attentional issues may go unreported or remain elusive. Consequently, assessing adults with hearing loss regarding attention, concentration, and fatigue using current evaluation methodologies poses challenges in arriving at definitive conclusions.

Hearing aids, frequency-modulated systems and auditory rehabilitation may be useful options for managing fatigue in patients with hearing loss. However, no randomised controlled intervention studies have been published for single-sided deafness patients before and after hearing rehabilitation to reduce chronic fatigue.

## Conclusion

We showed that individuals with single-sided deafness have more subjective fatigue and weaker performance in concentration and motivation but average performance in activity. Low concentration performance is related to poor cognitive ability. Duration of deafness is not correlated with fatigue severity. We propose that aetiology differences may have contributed to fatigue level severity irrespective of deafness duration. Subjective fatigue, concentration, and motivation should be considered after hearing rehabilitation for individuals with single-sided deafness.

## Data Availability

The datasets generated and/or analysed during the current study are available from the corresponding author upon reasonable request.
